# Chemical, Antioxidant and Antimicrobial Investigations of *Pinus cembra* L. Bark and Needles

**DOI:** 10.3390/molecules16097773

**Published:** 2011-09-13

**Authors:** Cristina Lungu Apetrei, Cristina Tuchilus, Ana Clara Aprotosoaie, Adrian Oprea, Karl Egil Malterud, Anca Miron

**Affiliations:** 1 Department of Plant and Animal Biology, School of Pharmacy, Gr. T. Popa University of Medicine and Pharmacy, Universitatii Street Number 16, 700115 Iasi, Romania; E-Mail: ape3c@yahoo.com (C.L.A.); 2 Department of Microbiology, School of Medicine, Gr. T. Popa University of Medicine and Pharmacy, Universitatii Street Number 16, 700115 Iasi, Romania; E-Mail: ctuchilus@yahoo.com (C.T.); 3 Department of Pharmacognosy, School of Pharmacy, Gr. T. Popa University of Medicine and Pharmacy, Universitatii Street Number 16, 700115 Iasi, Romania; E-Mail: anaclara70@yahoo.com (A.C.A.); 4 Institute of Biological Research, Lascar Catargi Street Number 47, 700107 Iasi, Romania; E-Mail: a_aoprea@yahoo.co.uk (A.O.); 5 Department of Pharmaceutical Chemistry, School of Pharmacy, University of Oslo, P.O. 1068, Blindern, 0316 Oslo, Norway; E-Mail: k.e.malterud@farmasi.uio.no (K.E.M.)

**Keywords:** *Pinus cembra* L., polyphenols, antioxidant activity, antimicrobial activity

## Abstract

The chemical constituents and biological activity of *Pinus cembra* L. (Pinaceae), native to the Central European Alps and the Carpathian Mountains, are not well known. The aim of the present work was to examine the phenolic content, antioxidant and antimicrobial effects of hydromethanolic extracts of *Pinus cembra* L. bark and needles. Bark extract had higher concentrations of total phenolics (299.3 *vs.* 78.22 mg gallic acid equivalents/g extract), flavonoids (125.3 *vs.* 19.84 mg catechin equivalents/g extract) and proanthocyanidins (74.3 *vs.* 12.7 mg cyanidin equivalents/g extract) than needle extract and was more active as a free radical scavenger, reducing agent and antimicrobial agent. The EC_50_ values in the 2,2-diphenyl-1-picrylhydrazyl (DPPH), 2,2'-azino-bis(3-ethylbenzo-thiazoline-6-sulfonic acid) diammonium salt (ABTS) and reducing power assays were 71.1, 6.3 and 26 μg/mL for bark extract and 186.1, 24 and 104 μg/mL for needle extract, respectively. In addition, needle extract showed ferrous ions chelating effects (EC_50_ = 1,755 μg/mL). The antimicrobial effects against *Staphylococcus aureus*, *Sarcina lutea*, *Bacillus cereus*, *Escherichia coli*, *Pseudomonas aeruginosa* and *Candida albicans* were assessed by the agar diffusion method. Both extracts (4 mg/well) were active against all the microorganisms tested; bark extract showed higher inhibition on all strains. These results indicate that *Pinus cembra* L. bark and needles are good sources of phytochemicals with antioxidant and antimicrobial activities.

## 1. Introduction

*Pinus cembra* L. (Pinaceae; variously known as Swiss stone pine, Arolla pine, ceder pine, cembran pine) is a coniferous species growing in the Central European Alps and the Carpathian Mountains [1]. Several studies have investigated the knotwood part, buds, needles and cones of cembran pine [2,3,4,5,6,7]. Pinosylvin, pinosylvin monomethyl ether and dihydropinosylvin monomethyl ether were the main stilbenes identified in knotwood extract. In free radical trapping capacity assays (chemiluminescence based-methods) the stilbene extract (IC_50_ 0.84 μg/L) was shown to be a more effective scavenger of superoxide radicals than butylated hydroxyanisole (IC_50_ 2.7 μg/L) and Trolox (IC_50_ 6.3 μg/L), but it had a lower peroxyl radical scavenging capacity than Trolox (trapping capacity 4.2 mmol/g and 8.0 mmol/g, respectively). In *tert*-butylhydroperoxide induced lipid peroxidation in rat liver microsomes the stilbene extract (IC_50_ 132 μg/L) was more active than butylated hydroxyanisole (IC_50_ 198 μg/L), but less active than Trolox (IC_50_ 5.0 μg/L) [2]. Cembran pine knotwood also contains pinocembrin, a flavonoid which showed a weak lipid peroxidation inhibition potency (IC_50_ 1,135 μg/L) and a low peroxyl radical trapping capacity (0.49 mmol/g) [3]. Buds and young needles of cembran pine were reported to contain 3-*O*-glucosides of kaempferol and isorhamnetin and mono- and di-coumaryl glucosides of kaempferol [4]. Quinic and shikimic acids have also been reported as needle constituents [5]. In the male cones three major anthocyanins have been identified: Delphinidin-3-glucoside, cyanidin-3-glucoside and peonidin-3-glucoside [6]. Gas chromatography-mass spectrometry analysis of volatiles emitted by cones and foliage revealed the presence of several major monoterpenes (α-pinene, β-pinene, unseparated limonene and β-phellandrene, camphene, sabinene, myrcene) together with traces of terpinolene and bornyl acetate. Traces of tricyclene were also identified in cone volatiles. Cone oleoresin was reported to contain monoterpenes similar to those found in cone volatiles and sesquiterpenes (caryophyllene, humulene) [7].

Cembran pine grows at high altitude (from 800 m in the Carpathian Mountains to 2,500 m in the Piemontese Alps) being permanently exposed to stress factors such as low temperatures, elevated ozone levels and irradiance [1]. Both ozone exposure and irradiance are known to cause an increase of reactive oxygen species (ROS) resulting in tissue injury and cell death. In response to oxidative stress, plants react by increasing their production of enzymatic and non-enzymatic antioxidants. Wieser *et al.* [8] investigated the responses of cembran pine needles to controlled exposure to ozone free air, ambient and two-fold ambient ozone concentrations. Ozone exposure did not affect the amount of photosynthetic pigments (chlorophylls, carotenoids) and antioxidants (ascorbate, glutathione, α-tocopherol) in current season needles. Only one-year-old needles were affected by ambient ozone levels: total glutathione content significantly decreased together with a marked shift towards the oxidized form while the de-epoxidation state of the xanthophyll cycle pigments increased. These minor biochemical changes induced by ozone exposure indicate that *Pinus cembra* L. is well adapted to high altitude stress conditions [8].

Polyphenols are plant secondary metabolites which are able to reduce ROS by donating hydrogen atoms of phenolic hydroxyls and by transfering electrons from phenolic hydroxyls or phenoxide anions [9]. High levels of antioxidant polyphenols might explain, at least in part, the resistance of cembran pine to high altitude environmental conditions. In addition, many polyphenols act as antimicrobial agents mainly due to their ability to complex with proteins leading to loss of protein function. Thus, polyphenols inactivate surface-exposed adhesins, cell envelope transport proteins, membrane-bound enzymes but also intracelular enzymes causing alterations both in membrane permeability and in metabolic pathways. Polyphenols may also disrupt microbial membranes by interacting with membrane lipids [10,11]. Since polyphenols are known to occur in conifer barks and needles, this study aimed to evaluate the antioxidant and antimicrobial activities of *Pinus cembra* L. bark and needles in relation to the phenolic content.

## 2. Results and Discussion

### 2.1. Total Phenolic, Flavonoid and Proanthocyanidin Contents

Polyphenols are a class of secondary metabolites which play a key role as antioxidants; they are able to scavenge ROS which are produced in plant cells as a consequence to UV light and ozone exposure. Besides, polyphenols are known to have many other functions (defence against herbivores and pathogens, repellence of insects, attraction of pollinators) [12,13]. Among polyphenols, flavonoids and proanthocyanidins have attracted considerable interest due to their broad spectrum of biological effects (antioxidant, anti-inflammatory, vasorelaxant, antimicrobial, antiviral, anticarcinogenic, antimutagenic) [14,15]. Therefore, total phenolics, flavonoids and proanthocyanidins were quantified in cembran pine extracts. As it can be seen from the data given in Table 1, the total phenolic content was almost four times higher in bark extract than in needle extract. Likewise, flavonoid and proanthocyanidin contents were significantly higher in bark extract than in needle extract.

### 2.2. DPPH Radical Scavenging Assay

High levels of ROS induce oxidative damage to lipids, proteins, carbohydrates and DNA leading in humans to the so-called oxidative-stress related diseases (atherosclerosis, rheumatoid arthritis, diabetes mellitus, muscular dystrophy, cataracts, cancer) [14,16]. Antioxidants prevent or delay the onset and slow the progression of the above mentioned diseases. Despite a high activity, synthetic antioxidants induce toxic side effects and therefore the search for naturally occuring antioxidants has been greatly intensified in recent years [17,18]. In this respect, plant polyphenols have been reported to possess potent antioxidant effects [9,19,20]. Although cembran pine is well adapted to high altitude oxidative stress, the antioxidant potential of its bark and needles has not been investigated. As oxidative stress is a very complex process, several assays were used in studying the antioxidant effects of cembran pine extracts.

The 2,2-diphenyl-1-picrylhydrazyl (DPPH) assay is a simple, rapid and sensitive method for evaluating free radical scavenging ability. DPPH radical is purple-coloured and therefore it absorbs strongly at 517 nm. In the presence of hydrogen-donating antioxidants, DPPH radical is reduced to yellow-coloured diphenylpicrylhydrazine (DPPH-H), leading to a decrease in the 517 nm absorbance [21,22].

**Figure 1:**
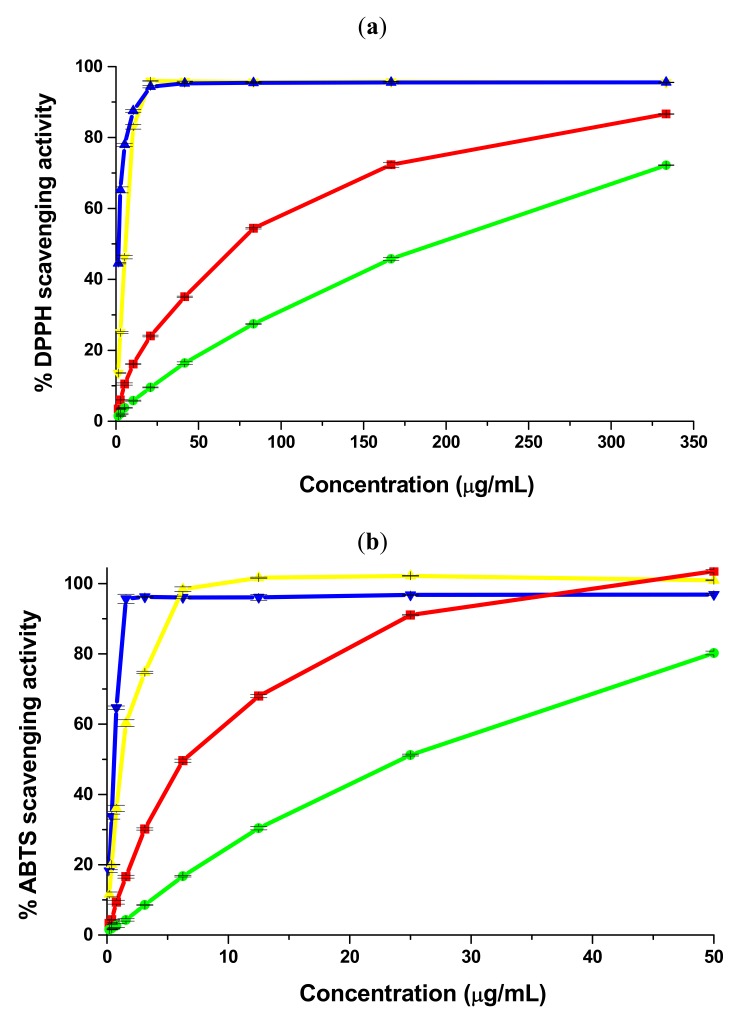

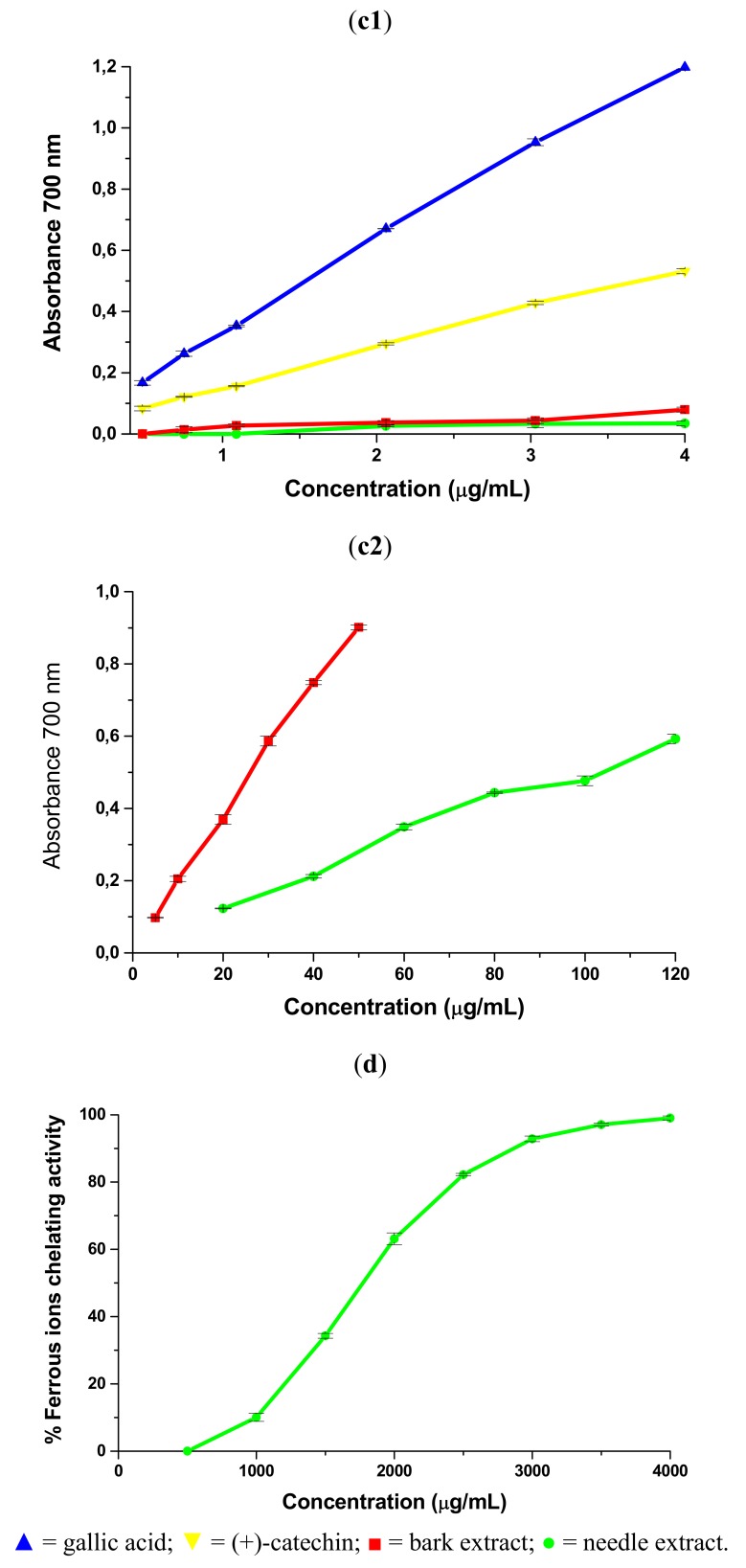
Antioxidant effects of *Pinus cembra* L. extracts assessed by several *in vitro* assays. (**a**) DPPH radical scavenging assay; (**b**) ABTS radical cation scavenging assay; (**c**) Reducing power assay; (**d**) Ferrous ion chelating ability assay.

As shown in Figure 1(a), both cembran pine extracts scavenged DPPH radical in a concentration-dependent manner. The scavenging activity of bark and needle extracts increased significantly from 3.4 ± 0.1% and 1.5 ± 0.2%, respectively (at 1.3 μg/mL) to 86.7 ± 0.1% and 72.2 ± 0.1%, respectively (at 333.33 μg/mL). DPPH scavenging effects of positive controls increased dose dependently up to 20.83 μg/mL. According to the EC_50_ values, bark extract showed a stronger DPPH scavenging activity than needle extract. The activities of both extracts were, however, inferior to those of gallic acid and (+)-catechin (Table 2) but are comparable to or better than those reported for some other plant extracts tested in the same assay under the same conditions. Wangensteen *et al*. investigated DPPH scavenging activity of several plant extracts and reported IC_50_ values higher than 200 μg/mL for extracts of *Coriandrum sativum* L. and *Sarcolobus globosus* Wall [23,24]. However, there are also reports on other plant extracts showing higher DPPH scavenging activity in this assay with IC_50_ values of 5.3–17 μg/mL [25,26]. In light of these literature data it appears that bark extract and, to a lesser extent, needle extract are reasonably efficient DPPH radical scavengers.

### 2.3. ABTS Radical Cation Scavenging Assay

This assay was also used to evaluate hydrogen-donating abilities of cembran pine extracts. 2,2'-Azino-bis(3-ethylbenzothiazoline-6-sulfonic acid) diammonium salt (ABTS) radical cation has a high absorbance at 734 nm. Hydrogen-donating antioxidants reduce the radical to the stable form ABTS-H leading to a decrease in absorbance at 734 nm [19,27]. ABTS scavenging activities of both extracts were significant and increased with their concentrations [Figure 1(b)]. At the highest concentration (50 μg/mL) bark extract completely eliminated the radical while needle extract showed 80.2 ± 0.5% scavenging activity. The EC_50_ values and the Trolox equivalent antioxidant capacity (TEAC) values suggested a higher ABTS scavenging activity for bark extract. Bark extract had a lower EC_50_ value and a higher TEAC value compared with needle extract (Table 2). At 3.12 μg/mL (+)-catechin scavenged ABTS radical cation by 74.7 ± 0.3%; at concentrations of 6.25-50 μg/mL (+)-catechin completely scavenged the radical. Gallic acid was more active than (+)-catechin; only 1.56 μg/mL gallic acid was needed for 95.7 ± 1.3% scavenging activity. The EC_50_ values and the TEAC values indicated that both positive controls were more active than cembran pine extracts (Table 2).

The improved ABTS radical cation decolorization assay developed by Re *et al.* [19] is one of the most widely used assays for the screening of antioxidant activity. Therefore, the literature abounds with reports of ABTS scavenging effects of plant extracts. Because of different ways of expressing antioxidant activity results [27,28,29,30,31,32], in many cases, a direct comparison of our results with other studies is not feasible. Nevertheless, it should be pointed out that, under similar experimental conditions, TEAC values (expressed as μM Trolox equivalent to 1 μg/mL antioxidant) of ascorbic acid and grape seed extract (GSE) were found to be 5.73 and 7.01, respectively [30]. As GSE and ascorbic acid are very strong antioxidants, it is obvious that both extracts, mainly bark extract having a TEAC value of 0.90 ± 0.01, have good ABTS scavenging effects.

### 2.4. Reducing Power Assay

Besides hydrogen donation, another important mechanism by which polyphenols scavenge ROS is the electron transfer. Antioxidants with electron-donating abilities reduce ferricyanide to ferrocyanide; ferrocyanide is then quantified as Perl's Prussian Blue at 700 nm [31,33]. Within this assay, EC_50_ values are the effective concentrations at which the absorbance is 0.5 [34]. Reducing powers of cembran pine extracts, gallic acid and (+)-catechin were studied within the concentration range of 0.48–4 μg/mL. As it can be seen from the dose-response curves depicted in Figure 1(c1), reducing powers of cembran pine extracts were low in this concentration range. Positive controls showed high activity. At 4 μg/mL gallic acid had a reducing power value of 1.2 ± 0.0 while reducing power of (+)-catechin reached 0.53 ± 0.00. In order to calculate EC_50_ values, higher concentrations of bark extract (5–50 μg/mL) and needle extract (20–120 μg/mL) were tested. Bark extract proved to be a stronger reducing agent than needle extract. Reducing power of bark extract increased from 0.1 ± 0.0 at 5 μg/mL to 0.9 ± 0.0 at 50 μg/mL. At 20 μg/mL needle extract had a reducing power value of 0.12 ± 0.00 which increased to 0.59 ± 0.01 at 120 μg/mL [Figure 1(c2)]. EC_50_ values of gallic acid and (+)-catechin were lower than those of bark and needle extracts indicating higher reducing activities for the positive controls (Table 2).

According to the absorbance values at 700 nm and EC_50_ values, cembran pine extracts were more effective than extracts of some wild edible mushrooms (*Lactarius deliciosus* (L.) Gray, *Tricholoma portentosum* (Fr.) Quél.) [34] but less effective than extracts of *Ocimum basilicum* L. [35]. Overall, bark extract proved to be a good reducing agent with stronger effects than needle extract.

### 2.5. Ferrous Ion Chelating Ability Assay

Another basic mechanism of antioxidant activity is ferrous ion chelation. Although iron is an essential element for living organisms, excess iron may lead to higher levels of ROS. In aqueous media ferrous ions generate ROS (superoxide anion radical, hydrogen peroxide) by autoxidation. In addition, ferrous ions are involved in conversion of superoxide anion radical and hydrogen peroxide into more reactive hydroxyl radical (Haber-Weiss reaction, Fenton reaction). As these ROS may oxidize different cell components (lipids, proteins, DNA), ferrous ion chelation can afford protection against oxidative damage [36].

The ferrous ion chelating ability was estimated by measuring the decrease in ferrozine-ferrous ions complex formation in the presence of cembran pine extracts. Ferrozine-ferrous ions complex absorbs at 562 nm. A chelator reduces the complexation of ferrous ions with ferrozine and consequently the absorbance at 562 nm decreases [37,38]. In this assay needle extract chelated ferrous ions in a concentration-dependent manner [Figure 1(d)] while bark extract, gallic acid and (+)-catechin showed no chelating effects. At 500 μg/mL needle extract showed no chelating effects, whereas at 4,000 μg/mL the chelating activity was 99.03 ± 0.59%. The EC_50_ value of needle extract is shown in Table 2. Analysis of literature data showed that ferrous ions chelation of needle extract is comparable to or lower than that of other plant extracts tested in similar experimental conditions [38].

Despite the hydrogen and electron donating abilities of cembran pine extracts which proved to be directly proportional to the concentration of total phenolics, the ferrous ion chelating effects did not correlate with the phenolic content. There are two possible explanations for this lack of correlation. First, chemical structure of polyphenols, namely certain structural features such as number and position of phenolic hydroxyl groups, have a significant influence on chelating activity [39]. Second, other compounds than phenolics may be involved in chelating potency of needle extract. There are reports on lack of correlation between total phenolic content and chelating capacity of vegetal extracts suggesting that non-phenolic compounds are mainly responsible for chelating metal ions. In addition, polysaccharides, peptides, proteins, oleoresins and saponins have been reported to chelate ferrous ions [40,41].

### 2.6. Agar Diffusion Method

The increasing incidence of infectious diseases, the severe side effects of many antibiotics, the development of antibiotic resistance justify the growing interest in the identification of new antimicrobial agents, both natural and synthetic [42]. Cembran pine has a significant resistance against pathogens, especially against the fungal parasite *Cronartium ribicola* J. C. Fish. ex Rabenh [43]. As phenolic metabolites are involved in resistance of trees to pathogens [12], the effects of cembran pine extracts against some human pathogenic microorganisms were also evaluated.

Table 3 presents the antimicrobial effects of cembran pine extracts. Both extracts (4 mg/well) were effective against all tested bacterial strains. Bark extract developed larger zones of inhibition than needle extract. Bark extract showed a good activity against *Sarcina lutea* ATCC 9341 and *Escherichia coli* ATCC 25922; its zones of inhibition were comparable to those of ampicillin (25 μg/disc). In addition, both extracts were active against *Pseudomonas aeruginosa* ATCC 27853. Bark extract produced a larger zone of inhibition than chloramphenicol (30 μg/disc); the inhibition zone of needle extract was comparable to that of chloramphenicol. Both extracts showed a significant activity against Gram-negative bacterial strains (*Escherichia coli* ATCC 25922, *Pseudomonas aeruginosa* ATCC 27853). This activity is important as Gram-negative bacteria are more resistant than Gram-positive bacteria due to lipopolysaccharide-rich outer membrane which significantly reduces the intracellular penetration of antibiotics [44].

Cembran pine extracts (4 mg/well) showed good activities against *Candida albicans* ATCC 10231. The inhibition zone developed by bark extract was larger than that of needle extract, but ca. 6 mm smaller than that of nystatin (100 μg/disc).

## 3. Experimental

### 3.1. Plant Material

Bark and needle samples of cembran pine were collected in Calimani Mountains, Romania in March and July 2008, respectively. The sampling site, located at approximately 1,450 m altitude, is characterized by an average annual rainfall of 1,022 mm, an average annual temperature of 1.8 °C–0.0 °C and annual thermal amplitudes between 15.1 °C and 24.9 °C. A full-grown tree was randomly selected for collection. Old needles were sampled from the same tree as the bark. The plant material was identified and authenticated by specialists from Anastasie Fatu Botanical Garden, Iasi, Romania. After collection, bark and needle samples were air-dried in a dark room at 23 ± 2 °C. Herbarium voucher samples are deposited in the Department of Pharmacognosy, Faculty of Pharmacy, Gr. T. Popa University of Medicine and Pharmacy, Iasi, Romania.

### 3.2. Chemicals

Gallic acid, (+) catechin hydrate, 2,2-diphenyl-1-picrylhydrazyl radical (DPPH), 2,2'-azinobis(3-ethylbenzothiazoline-6-sulfonic acid) diammonium salt (ABTS), (*R*)-(+)-6-hydroxy-2,5,7,8-tetramethylchroman-2-carboxylic acid (Trolox), potassium ferricyanide, iron (III) chloride were purchased from Sigma-Aldrich (Steinheim, Germany). Folin-Ciocalteu's phenol reagent and Mueller Hinton broth and agar were from Merck (Darmstadt, Germany). Trichloroacetic acid, ammonium iron (III) sulfate dodecahydrate and sodium nitrite were from Riedel-de-Haën (Sigma-Aldrich Laborchemikalien GmbH, Seelze, Germany). Sabouraud 4% glucose agar was from Fluka Biochemika (Switzerland). The antibiotic discs were purchased from Himedia (Mumbai, India). All other solvents and reagents were of analytical grade.

### 3.3. Microorganisms

The antimicrobial activity was studied using Gram-positive bacteria (*Staphylococcus aureus* ATCC 25923, *Sarcina lutea* ATCC 9341, *Bacillus cereus* ATCC 14579), Gram-negative bacteria (*Escherichia coli* ATCC 25922, *Pseudomonas aeruginosa* ATCC 27853) and pathogenic yeasts (*Candida albicans* ATCC 10231). All these strains were obtained from the Culture Collection of the Department of Microbiology, Gr. T. Popa University of Medicine and Pharmacy, Iasi, Romania.

### 3.4. Extraction

Dried bark and needles were finely ground in a knife mill and sieved to select particles smaller than 0.8 mm. Powdered bark and needles (20 g each) were mixed with 80% aqueous methanol (200 mL) and stirred by magnetic stirrer (500 rpm) for 1 h at room temperature. The extraction was repeated twice. Extracts were filtered under vacuum, combined, evaporated under reduced pressure at 40 °C and freeze-dried to afford 2.99 g bark extract and 3.01 g needle extract, respectively.

### 3.5. Total Phenolic Content

The total phenolic content of cembran pine extracts was determined using the Folin-Ciocalteu method [23,45]. Extracts were dissolved in dimethylsulfoxide (bark extract: 1 mg/mL; needle extract: 5 mg/mL). A sample (0.04 mL) was mixed with ultrapure water (3.16 mL) followed by addition of Folin-Ciocalteu's phenol reagent (0.2 mL). After 5 min, 20% sodium carbonate (0.6 mL) was added. The solutions were thoroughly mixed and incubated at room temperature for 2 h. The absorbance was then measured at 765 nm. A gallic acid calibration curve was plotted. The total phenolic content was expressed as gallic acid equivalents (mg gallic acid/g extract).

### 3.6. Total Flavonoid Content

The total flavonoid content was determined according to Ozsoy *et al.* [46]. An aliquot (0.25 mL) of each extract solution in dimethylsulfoxide was mixed with ultrapure water (1.25 mL) and 5% sodium nitrite (0.075 mL). The mixture was allowed to stand for 6 min followed by the addition of 10% aluminium chloride (0.15 mL). After 5 min, 1 M sodium hydroxide (0.5 mL) and ultrapure water (0.275 mL) were added sequentially. The mixture was shaken vigorously and the absorbance was measured immediately at 510 nm. The total flavonoid content was determined with a calibration curve using (+)-catechin hydrate as a reference compound. The results were expressed as (+)-catechin equivalents (mg (+)-catechin/g extract).

### 3.7. Total Proanthocyanidin Content

The total proanthocyanidin content was estimated by the method of Porter *et al.* [47]. Bark and needle extracts were dissolved in methanol in concentrations of 1 mg/mL and 2 mg/mL, respectively. Briefly, each extract solution in methanol (0.5 mL) was mixed with *n*-butanol-hydrochloric acid reagent (95:5, v/v, 3.0 mL) followed by the addition of ferric reagent (2% ferric ammonium sulfate dodecahydrate in 2 N hydrochloric acid, 0.1 mL). The solution was vortexed and then kept in a water bath at 95 °C for 40 min. After cooling, the absorbance was measured at 550 nm. The results were expressed as cyanidin equivalents (mg cyanidin/g extract) using ε = 17,360 L·mol^−1^·cm^−1^ and MW = 287.24 [48].

### 3.8. DPPH Radical Scavenging Assay

DPPH radical scavenging activity of cembran pine extracts was estimated using the method described by Malterud *et al.* [21]. Extracts were dissolved in dimethylsulfoxide in different concentrations ranging from 78.12 μg/mL to 20 mg/mL. Each dilution (0.05 mL) was mixed thoroughly with a solution of DPPH in methanol (2.95 mL, A_517nm_ = 1.00 ± 0.05). The absorbance of DPPH radical solution at 517 nm was measured before (A_start_) and 5 min after adding the extracts (A_end_). Gallic acid and (+)-catechin hydrate were used as positive controls. The ability to scavenge DPPH radical was calculated using the following formula:

DPPH radical scavenging activity (%) = 100 × (A_start_ − A_end_) / (A_start_)

### 3.9. ABTS Radical Cation Scavenging Assay

The assay was performed as described by Re *et al.* [19]. ABTS radical cation was generated by incubation of ABTS (7 mM) and potassium persulfate (2.45 mM) in the dark at room temperature for 16 h. The solution was further diluted with ethanol to an absorbance of 0.70 ± 0.02 measured at 734 nm and equilibrated at 30 °C. Extracts were dissolved in ethanol:water 7:3 (v/v) in different concentrations ranging from 19.53 μg/mL to 5 mg/mL. Each dilution (0.02 mL) was mixed with ABTS radical cation solution in a total volume of 2 mL. The absorbance at 734 nm was recorded 6 min after mixing. Gallic acid and (+)-catechin hydrate served as positive controls. The scavenging activity was calculated as:

ABTS radical cation scavenging activity (%) = 100 × (A_control_ − A_sample_) / (A_control_)

where A_control_ is the absorbance of the control and A_sample_ is the absorbance in the presence of extracts or positive controls. In order to calculate TEAC values (Trolox equivalent antioxidant capacity) Trolox was used as standard. The percentage of absorbance decrease *vs.* Trolox concentration (μM) was plotted. For extracts and positive controls there were selected three concentrations (μg/mL) producing a percentage of absorbance decrease in the most linear region of Trolox curve (20%–80% activity). Percentage of absorbance decrease as a function of selected concentrations was plotted for each extract and positive control. TEAC (μM concentration of Trolox equivalent to 1 μg/mL extract/positive control) was calculated as the ratio between the slopes of dose-response curves of extracts/positive controls and Trolox [19,30].

### 3.10. Reducing Power Assay

The reducing power was estimated using the method of Oyaizu as previously described [31,33]. Extracts in 0.2 M phosphate buffer, pH = 6.6 (2.5 mL; bark extract: 0.08–8.25 mg/mL; needle extract: 0.08–19.80 mg/mL) were mixed with 1% potassium ferricyanide (2.5 mL). The mixture was incubated at 50 °C for 20 min. The reaction was stopped by adding 10% trichloroacetic acid (2.5 mL) followed by centrifugation at 3,000 rpm for 10 min. A volume of the upper layer (2.5 mL) was mixed with ultrapure water (2.5 mL) and 0.1% ferric chloride (0.5 mL). After 90 s the absorbance of the mixture was measured at 700 nm. Gallic acid and (+)-catechin hydrate served as positive controls. A high absorbance of the reaction mixture indicated a high reducing capacity.

### 3.11. Ferrous Ion Chelating Ability Assay

The assay used to determine ferrous ions chelating capacity of cembran pine extracts was based on the method of Dinis *et al.* [37] with minor changes [38]. The reaction mixture contained different concentrations of extracts (0.40 mL, 2.5–20 mg/mL), methanol (1.480 mL) and 2 mmol/L ferrous chloride (0.04 mL). Ferrozine (0.08 mL, 5 mmol/L) was further added followed by vigorous mixing. After 10 min the absorbance of the ferrozine-ferrous ions complex was measured at 562 nm. Gallic acid and (+)-catechin hydrate were used as positive controls. The percentage of ferrous ions chelating activity was calculated using the formula:

Ferrous ion chelating activity (%) = 100 × (A_control_ − A_sample_) / (A_control_)

where A_control_ is the absorbance of the control and A_sample_ is the absorbance in the presence of extracts or positive controls.

### 3.12. Agar Diffusion Method

Antimicrobial activity of cembran pine extracts was evaluated by the agar well diffusion method according to described protocols [49,50]. A small amount of each microbial culture was diluted in sterile 0.9% NaCl until the turbidity was equivalent to McFarland standard no. 0.5 (10^6^ CFU/mL). The suspensions were further diluted 1:10 in Mueller Hinton agar for bacteria and Sabouraud agar for yeasts and then spread on sterile Petri plates (25 mL/Petri plate). Sterile stainless steel cylinders (5 mm internal diameter; 10 mm height) were applied on the agar surface in Petri plates. Each extract (4 mg; 0.2 mL of 20 mg/mL in dimethylsulfoxide) was added to each cylinder. Commercial available discs containing ampicillin (25 μg/disc), chloramphenicol (30 μg/disc) and nystatin (100 μg/disc) were also placed on the agar surface. The plates were incubated at 37 °C for 24 h (bacteria) and at 24 °C for 48 h (yeasts). After incubation the diameters of inhibition zones were measured.

### 3.13. Statistical Analysis

All assays were carried out in triplicate. Results are expressed as means ± SD. The EC_50_ values were calculated by linear interpolation between values above and below 50% activity.

## 4. Conclusions

Although cembran pine has high resistance to biotic and abiotic stress factors, no studies have investigated the antioxidant and antimicrobial potential of its bark and needles, and to our knowledge, this is the first such report. Although bark and needle extracts were less active than the positive controls, it is expected that further fractionation (especially of bark extract) will lead to highly active fractions and/or pure compounds with respect to antioxidant and antimicrobial activity.

In conclusion, *Pinus cembra* L. bark and needles contain phytochemicals of putative therapeutic interest with respect to antioxidant and antimicrobial effects. These compounds may have the possibility of serving as prototype structures for more potent derivatives. Studies on isolation of antioxidant and antimicrobial constituents of cembran pine bark and needles are in progress.

## Figures and Tables

**Table 1:** Total phenolic, flavonoid and proanthocyanidin contents in *Pinus cembra* L. extracts.

Extract	Total phenolic content *	Total flavonoid content **	Total proanthocyanidin content ***
Bark extract	299.3 ± 1.4	125.3 ± 1.2	74.3 ± 0.5
Needle extract	78.22 ± 0.44	19.84 ± 0.57	12.7 ± 0.3

* mg gallic acid/g extract; ** mg (+)-catechin/g extract; *** mg cyanidin/g extract.

**Table 2:** Effects of *Pinus cembra* L. extracts in different antioxidant assays.

Extract/Positive control	DPPH radical scavenging assay EC_50_ *	ABTS radical cation scavenging assay		Reducing power assay EC_50_ *	Ferrous ion chelating ability assay EC_50_ *
		EC_50_ *	TEAC **		
Bark extract	71.1 ± 0.5	6.3 ± 0.2	0.90 ± 0.01	26.0 ± 0.3	-
Needle extract	186.1 ± 1.7	24.0 ± 0.2	0.3 ± 0.0	104 ± 2	1,755 ± 22
Gallic acid	1.56 ± 0.05	0.6 ± 0.0	18.13 ± 0.16	1.53 ± 0.00	-
(+)-Catechin	5.56 ± 0.05	1.16 ± 0.05	7.92 ± 0.05	3.70 ± 0.03	-

* μg/mL; ** μM Trolox equivalent to 1 μg/mL extract/positive control.

**Table 3:** Antimicrobial effects of *Pinus cembra* L. extracts.

Extract/Positive control	Diameter of inhibition zone (mm)					
	*S. aureus* ATCC 25923	*S. lutea* ATCC 9341	*B. cereus* ATCC 14579	*E. coli* ATCC 25922	*Ps. aeruginosa* ATCC 27853	*C. albicans* ATCC 10231
Bark extract (4 mg/well)	19.33 ± 1.15	29.33 ± 1.15	16 ± 0	16.33 ± 0.57	15 ± 1	24.33 ± 0.57
Needle extract (4 mg/well)	14.66 ± 0.57	25.66 ± 0.57	15.33 ± 0.57	12.66 ± 0.57	12.66 ± 0.57	20 ± 0
Ampicillin (25 μg/disc)	29.33 ± 0.57	30.33 ± 0.57	n.z.	16.33 ± 0.57	n.z.	n.d.
Chloramphenicol (30 μg/disc)	30 ± 1	33 ± 0	27 ± 1	21.66 ± 1.15	12 ± 1	n.d.
Nystatin (100 μg/disc)	n.d.	n.d.	n.d.	n.d.	n.d.	30 ± 1

n.z.: no zone of inhibition; n.d.: not determined.
